# Does a coupling capacitor enhance the charge balance during neural stimulation? An empirical study

**DOI:** 10.1007/s11517-015-1312-9

**Published:** 2015-05-29

**Authors:** Marijn N. van Dongen, Wouter A. Serdijn

**Affiliations:** Section Bio-Electronics, Delft University of Technology, Mekelweg 4, 2628CD Delft, The Netherlands

**Keywords:** Electric stimulation, Implantable neurostimulators, Electrodes

## Abstract

Due to their DC-blocking characteristic, coupling capacitors are widely used to prevent potentially harmful charge buildup at the electrode–tissue interface. Although the capacitors can be an effective safety measure, it often seems overlooked that coupling capacitors actually introduce an offset voltage over the electrode–tissue interface as well. This work investigates this offset voltage both analytically and experimentally. The calculations as well as the experiments using bipolar-driven platinum electrodes in a saline solution confirm that coupling capacitors introduce an offset, while they barely contribute to the passive charge balancing. In particular cases, this offset is shown to reach potentially dangerous voltage levels that could induce irreversible electrochemical reactions. This work therefore suggests that when the use of coupling capacitors is required, the offset voltage should be analyzed for all operating conditions to ensure it remains within safe boundaries.

## Introduction

Neural stimulation is becoming an increasingly popular clinical treatment methodology for a wide variety of diseases. In most cases, an implantable pulse generator (IPG) is used to deliver stimulation pulses to electrodes that are placed in the target area. The safety of the device is of major concern, since a faulty stimulation signal can cause irreversible damage to the neural tissue. It is especially important to prevent the flow of DCs through the electrodes [3, 7].

The use of coupling capacitors between the stimulator and the electrodes is widely considered to be an effective safety mechanism [10, 11], and indeed, various advantages concerning the use of coupling capacitors have been identified [4]. The first important advantage is the prevention of DCs in the event of device failure [9]. If, for example, one of the electrodes shorts to the supply voltage, the coupling capacitor will prevent a prolonged DC current through the electrodes.

The second important advantage that is attributed to coupling capacitors is that they improve the performance of passive charge-balancing techniques [4, 14, 15]. Charge balancing is important for polarizable electrodes to keep the electrode–tissue interface within an electrochemically safe regime [7]. A coupling capacitor helps due to its high-pass characteristics, which limits the flow of DCs, and hence, no net charge can be injected into the tissue.

A disadvantage of coupling capacitors is that their required value is often too high to be integrated on an IC [15], and hence, they are realized using bulky external components. Many studies have focused on designing stimulator output stages with accurate charge-balancing circuits [8, 13] in order to eliminate the need of coupling capacitors. Others have proposed high-frequency operation to reduce their size [5]. Indeed, the results seem to suggest that the proposed mechanisms are good enough to prevent charge accumulation on the tissue even without coupling capacitors. However, it is not clear how these systems can guarantee safety in the event of a device failure. For this reason, many stimulator systems still require the use of coupling capacitors.

Although widely used, it often seems overlooked that a coupling capacitor eliminates control over the DC voltage across the electrodes. As will be shown in this work, it is therefore possible for an offset voltage *V*_os_ to develop over the electrode–tissue interface, even when the electrodes and capacitors are shorted in between the stimulation pulses and charge-balanced biphasic stimulation is used.

If *V*_os_ becomes too large, the electrode–tissue interface may leave the electrochemically safe regime, triggering the production of potentially dangerous reaction products. In this case, the intended safety mechanisms of the coupling capacitor create the opposite result: A potentially dangerous situation is created. In this work, the value of *V*_os_ is analyzed over various operating conditions, both analytically and experimentally. This gives insight in when *V*_os_ is exceeding a predefined safe regime.

## Methods

**Fig. 1:**
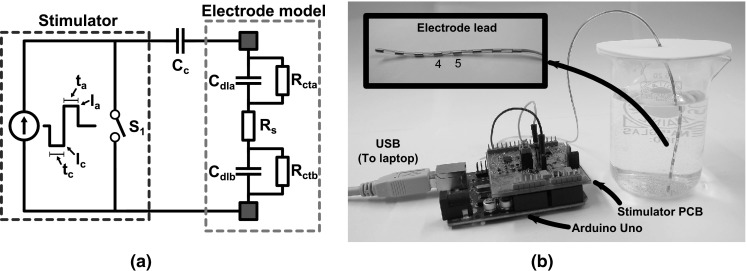
**a** A basic setup of a biphasic constant current stimulator system is shown that includes a coupling capacitor *C*_c_ and an electrode model. **b** A picture of the measurement setup is shown with a detail of the electrode lead where contacts *4* and *5*, which were used for stimulation, are indicated

A basic setup of a biphasic stimulator system is depicted in Fig. 1a: The coupling capacitor *C*_c_ is connected in series with the stimulator and the electrodes. The stimulation source in Fig. 1a is a biphasic constant current stimulator with a cathodic first stimulation pulse with amplitude *I*_c_ and duration *t*_c_. The anodic charge cancelation phase follows with amplitude *I*_a_ and duration *t*_a_. Most stimulator systems apply a passive charge-balancing scheme [15], in which the series connection of the electrodes and coupling capacitor are shorted after the stimulation cycle by closing switch *S*_1_ to discharge *C*_dl_. The duration of shorting *t*_dis_ is determined by the repetition rate *f*_stim_ = 1*/t*_stim_ of the stimulation, since *S*_1_ needs to be opened again when the next stimulation cycle starts.

As shown in Fig. 1a, the electrodes are modeled as a resistance *R*_s_ in series with capacitors (*C*_dla_ and *C*_dlb_) and resistors (*R*_cta_ and *R*_ctb_) that model the electrode–tissue interfaces of both electrodes [6]. The electrodes used in this study are single percutaneous octrode leads (manufactured by ANS, currently St. Jude Medical): They consist of eight ring-shaped platinum contacts that are distributed on a single lead. Each electrode has a diameter of 1.5 mm and a width of 3 mm (area 0.14 cm^2^). A picture of the electrodes is depicted in Fig. 1b. These types of electrodes are typically used for spinal cord stimulation, and the stimulation amplitudes used in this paper are based on the specifications of the EON™ IPG (also from St. Jude Medical) [16]. The electrodes were submerged in a phosphate-buffered saline (PBS) solution containing the following: 1.059 mM KH_2_PO_4_, 155.172 mM NaCl, 2.966 mM Na_2_HPO_4_–7H_2_O (pH 7.4, Gibco^®^ Life technologies™). The electrodes were connected in a bipolar fashion by selecting contacts 4 and 5 as the anode and cathode (see Fig. 1b). The other contacts were left floating.

Using an HP4194A impedance analyzer (excitation amplitude 0.1 V), it was found that for these electrodes in the PBS solution, *R*_s_ ≈ 100 Ω and *C*_dl_ ≈ 1.5 μF. Here, *C*_dl_ is the capacitive part of both electrode–tissue interfaces combined. The value of *R*_ct_ ≈ 1 MΩ (also combining both interfaces) was determined by measuring the voltage over the electrodes due to a 5-nA DC from a Keithley 6430 sub-femtoamp sourcemeter.

### Determining *V*_os_

After the anodic phase, both *C*_c_ and *C*_dl_ will be charged. Upon closing *S*_1_, these capacitors will be discharged with a time constant:

1 $$\tau_{\text{dis}} = R_{s} C_{\text{eq}} \quad C_{\text{eq}} = \frac{{C_{c} C_{\text{dl}} }}{{C_{c} + C_{\text{dl}} }}$$

If *S*_1_ would be closed sufficiently long, a pseudosteady state is reached in which:

2 $$V_{\text{Cc}} + V_{\text{Cdl}} = 0$$

Here, *V*_Cc_ is the voltage over *C*_c_. If *S*_1_ is closed even longer, *C*_dl_ will continue to discharge through *R*_ct_ with time constant *τ*_2_ = *R*_ct_*C*_dl_ until *V*_Cdl_ = 0 V and the actual steady state is reached. However, usually *t*_dis_ ≪ *τ*_2_, and therefore, only the pseudosteady state is reached.

Note that Eq. (2) does *not* guarantee that *V*_Cdl_ = 0 in pseudosteady state: It is an under-determined equation, and *V*_Cc_ = −*V*_Cdl_ can have any value. Only when both *C*_c_ and *C*_dl_ are ideal capacitors, the same current is flowing through both capacitors during a stimulation cycle, which causes *V*_Cc_ = *V*_Cdl_ = 0 V in pseudosteady state. If these requirements are not met (e.g., when *R*_ct_ ≠ ∞), the current though *C*_c_ does not equal to the current through *C*_dl_, which will cause *V*_Cdl_ = -*V*_Cc_ ≠ 0 in pseudosteady state. This charge imbalance can accumulate over many stimulation cycles, which creates an offset in *V*_Cdl_ [2].

**Fig. 2:**
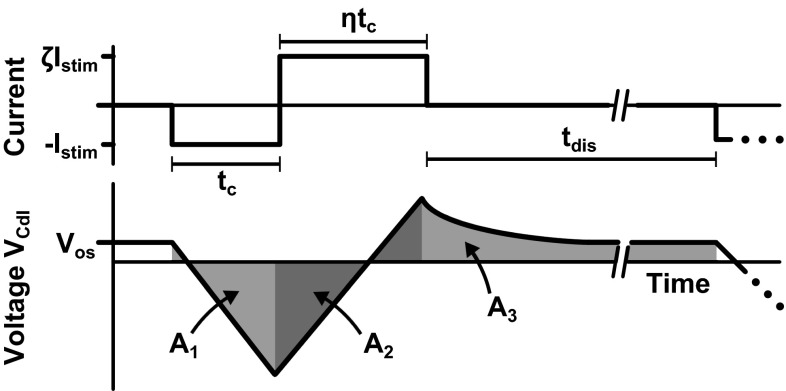
Schematic plot of *V*_Cdl_ during a biphasic stimulation cycle with charge mismatch. When *V*_os_ is stable, the area *A*_1_ + *A*_2_ + *A*_3_ equals zero

We refer to Fig. 2 to analyze *V*_Cdl_ when after many stimulation cycles the offset voltage *V*_os_ is stable. In order for this voltage to be stable, the average current through *R*_ct_ must be zero such that no charge is lost that causes an inequality in the charge accumulated on *C*_dl_ with respect to *C*_*c*_. Therefore, it must hold that the average value of *V*_Cdl_ (and hence the area as indicated in Fig. 2) is zero as well.

To find the value of *V*_os_ for which this requirement is met, it is assumed that the cathodic stimulation phase is characterized by a duration *t*_c_ and amplitude *I*_c_ = *I*_stim_. In the anodic phase, both the duration *t*_a_ = *ηt*_c_ and the amplitude *I*_a_ = *ζI*_stim_ can include mismatch. Furthermore, it is assumed that *t*_dis_ ≪ *τ*_2_ (such that pseudosteady state is reached) and that *R*_ct_ is large enough to be neglected in the analysis (but as stated above, it must be finite). The areas *A*_1_, *A*_2_ and *A*_3_ are found as:

3a $$A_{1} = \int_{0}^{{t_{\text{c}} }} {\left( {V_{\text{os}} - \frac{{I_{\text{stim}} t}}{{C_{\text{dl}} }}} \right)} {\text{d}}t = V_{\text{os}} t_{\text{c}} - \frac{{I_{\text{stim}} t_{{_{\text{c}} }}^{2} }}{{2C_{\text{dl}} }}$$

3b $$A_{2} = \int_{0}^{{\eta t_{\text{c}} }} {\left( {V_{\text{os}} - \frac{{I_{\text{stim}} t_{\text{c}} }}{{C_{\text{dl}} }} + \frac{{\zeta I_{\text{stim}} t}}{{C_{\text{dl}} }}} \right)} {\text{d}}t = V_{\text{os}} \eta t_{\text{c}} - \frac{{I_{\text{stim}} \eta t_{\text{c}}^{2} }}{{C_{\text{dl}} }} + \frac{{\zeta I_{\text{stim}} \left( {\eta t_{\text{c}} } \right)^{2} }}{{2C_{\text{dl}} }}$$

3c $$A_{3} = \int_{0}^{{t_{\text{dis}} }} {\left( {V_{\text{os}} - \left( {1 - \zeta \eta } \right)\frac{{I_{\text{stim}} t_{\text{c}} }}{{C_{\text{dl}} }}\exp \left( {\frac{ - t}{{R_{\text{s}} C_{\text{dl}} }}} \right)} \right)} {\text{d}}t = V_{\text{os}} t_{\text{dis}} - \left( {1 - \zeta \eta } \right)I_{\text{stim}} t_{\text{c}} R_{\text{s}}$$

By setting *A*_1_ + *A*_2_ + *A*_3_ = 0 and solving for *V*_os_, the following equation is obtained:

4 $$V_{\text{os}} = \frac{{(0.5 + \eta - 0.5\zeta \eta^{2} )I_{\text{stim}} t_{\text{c}}^{2} + (1 - \zeta \eta )I_{\text{stim}} C_{\text{dl}} t_{\text{c}} }}{{C_{\text{dl}} t_{\text{c}} (1 + \eta ) + t_{\text{dis}} }}$$

If *ζ* = *η* = 1, which means that perfectly charge-balanced stimulation is applied, the following equation holds:

5 $$V_{\text{os}} = \frac{{I_{\text{stim}} t_{\text{c}}^{2} }}{{C_{\text{dl}} (2t_{\text{c}} + t_{\text{dis}} )}} = \frac{{I_{\text{stim}} t_{\text{c}}^{2} }}{{C_{\text{dl}} (t_{\text{stim}} )}}$$

**Fig. 3:**
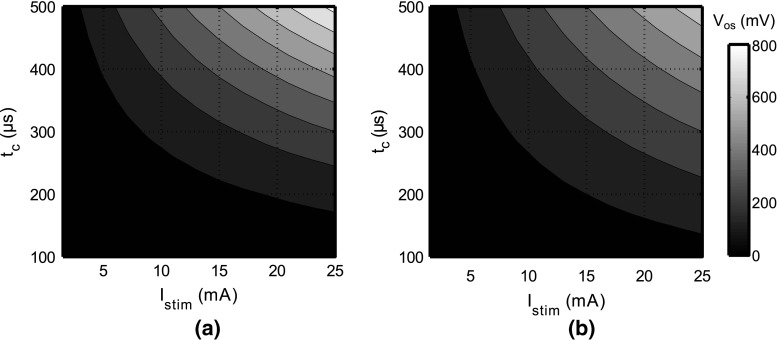
Overview of the pseudosteady-state offset voltages *V*_os_ for a variety of stimulation settings. **a** A perfectly charge-balanced stimulation waveform is chosen, and *V*_*os*_ is determined according to Eq. (5) with *f*_stim_ = 200 Hz. **b** A monophasic stimulation waveform is used, and *V*_os_ is determined using Eq. (4) with *η* = 0

In Fig. 3a, the value of *V*_os_ is depicted for a charge-balanced stimulation cycle with *f*_stim_ = 200 Hz. This cycle includes a coupling capacitor. For small *I*_stim_ and *t*_c_, the value of *V*_os_ is small and will have negligible influence on the system. However, for larger stimulation intensities, *V*_os_ starts to increase toward several hundreds of millivolts (up to 800 mV for the maximum intensity).

Equation (4) can also be used to analyze monophasic stimulation patterns by choosing *η* = 0. In Fig. 3b, the values of *V*_os_ are plotted for this situation. Somewhat surprisingly, these values are smaller than the biphasic charge-balanced stimulation. However, this can be explained by the fact that due to the relatively low value of *R*_s_, the discharge current during *t*_dis_ is larger than *I*_stim_, and hence, the electrodes discharge faster toward pseudosteady state as compared to the biphasic stimulation waveform.

### Verifying *V*_os_

To verify Eq. (4), the response of an electrode system was analyzed using both simulations and measurements in a saline bath. To simulate the response of these electrodes, the circuit from Fig. 1 was implemented in a simulator (LT-Spice). Switch *S*_1_ was chosen to have *R*_off_ = 10 MΩ to mimic the limited output impedance of the current source and *R*_on_ = 10 Ω. The stimulation current was chosen to be *I*_stim_ = 1.5 mA (*ζ* = 1), while an 8 % charge mismatch was introduced by making *t*_c_ = 460 μs and *t*_a_ = 500 μs (η = 1.087). After the stimulation cycle, switch *S*_1_ was closed for *t*_dis_ = 9 ms before the next stimulation pulse is started. This makes the stimulation repetition rate slightly higher than 100 Hz.

Using Eq. (1), it is found that *t*_dis_ > 60 *τ*_dis_, which means that *V*_Cdl_ and *V*_Cc_ can be assumed to have reached their pseudosteady-state values. Also *τ*_2_ = 1.5 s ≫ *τ*_dis_, which means that the system will stay in pseudosteady state and will not have the opportunity to fully discharge.

The value of *C*_c_ should be chosen well above *C*_dl_ in order to limit the contribution of *C*_c_ to the voltage headroom of the stimulator [15]. In this particular case, it was chosen to make *C*_c_ = 8.8 μF, based on the availability of components for the measurements. The circuit was simulated over many stimulation cycles (up to 200 s) to analyze the voltage over *C*_dl_ and *C*_c_. To minimize leakage introduced by the simulation setup, the minimum conductance of the SPICE simulator was lowered from *G*_min_ = 1 pΩ^−1^ to *G*_min_ = 1 fΩ^−1^. After a simulation, MATLAB was used to select the time stamps that correspond to pseudosteady state to obtain the values of *V*_os_ over many stimulation cycles.

**Fig. 4:**
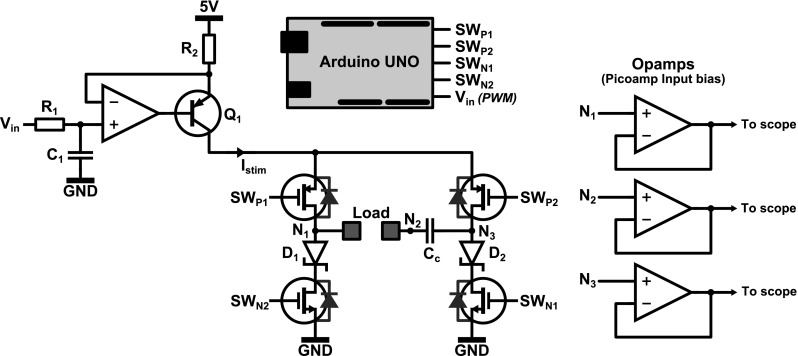
Measurement setup used to verify the influence of the coupling capacitor *C*_*c*_ on the charge cancelation. A constant current source implemented using *Q*_1_ is connected to the load via an H-bridge configuration (MOSFET switches), which allows bidirectional stimulation. An Arduino Uno is used for the control of the circuit, while buffers are used to prevent loading of the system during measurements

After simulations, a stimulation circuit was built using discrete components as depicted in Fig. 4. Transistor *Q*_1_ (2N3906) implements a current source together with resistor *R*_2_ and the opamp (LMV358). The output current *I*_stim_ is controlled using the PWM signal *V*_in_ that is filtered using *R*_1_ = 1 MΩ and *C*_1_ = 1 μF. Using the H-bridge topology implemented with MOSFET devices (NTZD3155C), the current can be injected bidirectionally through the load during the cathodic and anodic stimulation phase. An Arduino Uno is used to control the switches: During the cathodic phase, switches *SW*_P1_ and *SW*_N1_ are closed, while during the anodic phase, switches *SW*_P2_ and *SW*_N2_ are closed. The tissue is shorted in between the stimulation pulses by closing *SW*_P1_ and *SW*_P2_. Diodes *D*_1_ and *D*_2_ (CD0603–B00340) are needed to prevent unwanted current flow through the body diodes of *SW*_N1_ and *SW*_N2_: If *C*_dl_ is charged beyond 0.6 V during the cathodic phase, the body diodes of *SW*_N1_ and *SW*_N2_ otherwise become forward biased when the stimulation direction is reversed.

The Arduino was programmed with four different stimulation settings as summarized in Table 1. The first setup uses no coupling capacitor, and it is verified that *C*_dl_ is indeed charged back to 0 V after a stimulation cycle. The second setup uses a low-intensity stimulation cycle with a positive charge mismatch, while the third setup uses a high-intensity stimulation cycle (close to the maximum stimulation intensity possible before the current source would clip to the 5 V supply voltage). The fourth experiment uses a monophasic stimulation waveform. All measurements were taken after stimulation was enabled sufficiently long (at least 5 min) to allow the voltages to settle.

**Table 1:** Stimulation settings used during measurements

Nr.	Waveform	*I*_stim_ (mA)	*t*_c_ (μs)	Mismatch *η*	*f*_stim_ (Hz)	Incl. *C*_c_?
1	Biphasic	1.5	460	1.085 (*t*_a_ = 500 μs)	110	No
2	Biphasic	1.5	460	1.085 (*t*_*a*_ = 500 μs)	110	Yes
3	Biphasic	15	200	0.75 (*t*_*a*_ = 150 μs)	400	Yes
4	Monophasic	15	200	0	100	Yes

The load of the circuit in Fig. 4 first consisted of the electrode model from Fig. 1 (*R*_s_ = 100 Ω, *C*_dl_ = 1.5 μF, *R*_ct_ = 1 MΩ). Subsequently, the electrode model was replaced by the electrodes that were submerged in the PBS solution.

To measure the response of the system, the relevant output signals are buffered using picoampere input bias operational amplifiers (AD8625, powered with ±8 V) in order to prevent the measurement equipment from loading the system. It was found using simulations and measurements that a 10 MΩ ||12 pF standard probe largely distorts the measurement, as will be discussed further in the last section.

## Results

**Fig. 5:**
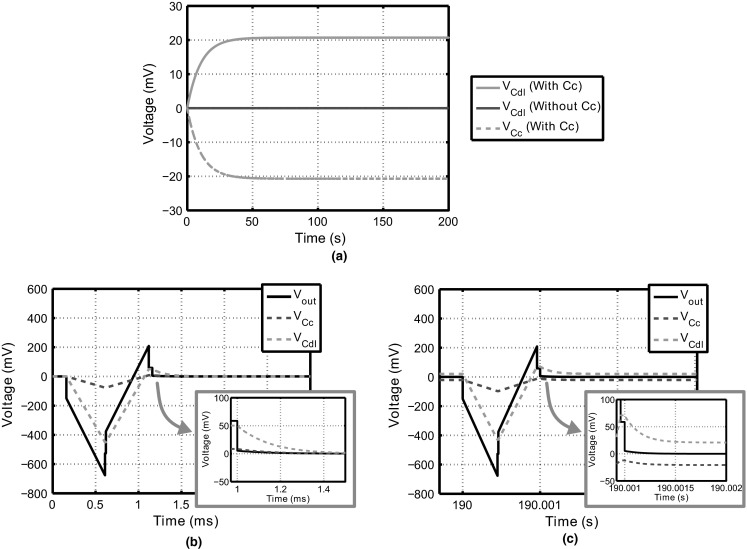
Simulation results of the circuit from Fig. 1. **a** The voltages *V*_Cdl_ and *V*_Cc_ are shown during the interval *t*_open_ over a large number of stimulation cycles. As can be seen, the coupling capacitor causes an offset. **b**, **c** The transient voltages are shown for the system with *C*_c_ and *R*_p_ = ∞ just after stimulation is initiated and after 190 s, respectively

Figure 5 shows the simulation results of the circuit from Fig. 1. In Fig. 5a, the value of *V*_Cdl_ in pseudosteady state is shown over many stimulation cycles. When no coupling capacitor is used, *V*_Cdl_ can discharge almost completely. When *C*_c_ is added in Fig. 5a, it is seen that after several stimulation cycles, *V*_Cdl_ = 20.7 mV. Indeed, the introduction of *C*_c_ causes an offset in *V*_Cdl_ in pseudosteady state. Furthermore, the simulated values correspond well with Eq. 4, which predicts *V*_os_ = 20.6 mV.

In Fig. 5b, c, the simulated transient behavior of the voltages in the circuit including *C*_c_ is shown for two time instances. Figure 5b shows the voltages right after the first stimulation cycle, while Fig. 5c shows the voltages during a stimulation cycle after 190 s of simulation time, where the offset is clearly visible.

**Fig. 6:**
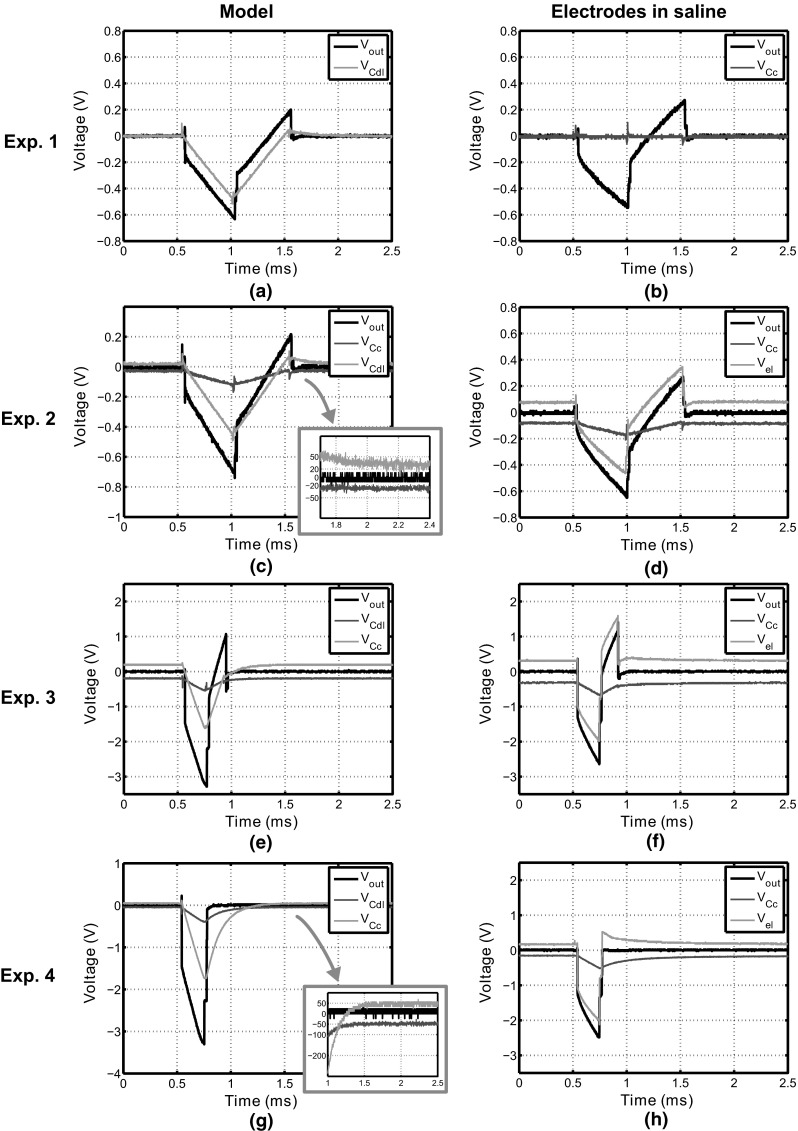
Measurement results from the experimental setup depicted in Fig. 4 with the load consisting of the electrode model (*left column*) and the electrodes in saline (*right column*). For both loads, four stimulation settings are used as described in Table 1. As can be seen, the offset voltage depends on the stimulation settings used, but is zero when no coupling capacitor is used (experiment 1)

In Fig. 6, the measurement results are presented for all experiments listed in Table 1. In all figures, *V*_out_ refers to the voltage measured over the output of the current source (between nodes *N*_1_ and *N*_3_ in Fig. 3) and *V*_el_ is the voltage over the electrode (nodes *N*_1_ and *N*_2_). For saline measurements, it is not possible to measure *V*_Cdl_ directly, and hence, *V*_el_ is shown instead.

## Discussion

The measured values for *V*_os_ are summarized in Table 2 and compared with the values calculated using Eq. (4). It is seen that the measurements with the model correspond well to the calculated values, indicating that the circuit implementation is working as expected. For the saline measurements, the values of *V*_os_ are higher than expected, and hence, the model underestimates the offset value introduced. This is most likely due to complex nonlinear behavior of the electrode–tissue interface that cannot be modeled using the simple capacitance *C*_dl_. The electrode model is a small-signal model (*C*_dl_ was found using a sinusoidal excitation of 0.1 V), and the measurement results show that the validity of the model is limited during a stimulation cycle. From these results and the plots in Fig. 6, we can draw three important conclusions.

**Table 2:** Calculated and measured values of *V*_os_ for the experiments summarized in Table 1

Experiment	Equation (4) (mV)	Measurement (model) (mV)	Measurement (saline) (mV)
1	0	0	0
2	21.6	25	80
3	201	200	320
4	50	50	165

1. First of all, coupling capacitors barely improve the way in which *V*_Cdl_ returns to equilibrium. The only way in which *C*_c_ contributes is by making *τ*_dis_ (Eq. 1) smaller during the *t*_dis_ interval [4]. This causes the interface to discharge toward equilibrium slightly faster. However, since *C*_c_ ≫ *C*_dl_, the influence on *τ*_dis_ is negligible, and hence, coupling capacitors barely improve the charge cancelation.
2. Second of all, coupling capacitors introduce an offset in the pseudosteady-state value of the electrodes. The value of *V*_os_ can be predicted using Eq. (4), although it was found that this equation underestimated the offset measured from the electrodes in saline.

The question is whether or not *V*_os_ introduces potential safety issues. For small values of *V*_os_, no problems are likely to occur: As long as no irreversible faradaic reactions are triggered, no harmful effects are to be expected. Even more so, *V*_os_ will increase the amount of charge that can be injected [1], because *V*_os_ reduces the peak voltage of *V*_Cdl_ during a stimulation cycle.

However, when *V*_os_ increases toward the threshold of irreversible faradaic reactions (600–900 mV for platinum electrodes [12]), problems can be expected. In this case, the interface is experiencing a significant offset voltage during the *t*_dis_ interval, during which irreversible reactions might occur. For high stimulation intensities, Fig. 3 predicts values of *V*_os_ that are close to or exceed the maximum safe voltage window.

3 Finally, secondary effects can have a strong effect on *V*_os_. Using the settings of experiment 2, measurements were repeated with both the model and the electrodes as load. This time, the voltages were not buffered using the picoampere input bias opamps, but 10 MΩ ||12 pF probes (referenced to ground) were connected to *N*_1_, *N*_2_ and *N*_3_ directly. This has a large impact on the offset voltage: It increases from 25 mV to 2 V (model) and from 80 mV to 0.6 V (saline).

All in all, it can be concluded that in contrast to what many other studies have suggested [4, 14, 15], the introduction of *C*_c_ does not improve the charge-balancing process and it is furthermore associated with the loss of control over the pseudosteady-state value of *V*_Cdl_. Instead of ensuring safety by returning the electrode interface voltage back to 0 V, the coupling capacitor introduces an unwanted offset in the interface voltage that is hard to control by the stimulator and, moreover, is sensitive to secondary effects.

Although this work suggests that coupling capacitors are not beneficial for charge cancelation purposes, they still protect the electrodes and tissue from DCs in case of a device or software failure. Depending on the application, this could require the need to still use these capacitors. In that case, the results from this study show that the stimulation settings should be limited to ensure that under all operating conditions, *V*_os_ does not exceed any predefined safety window.

It is possible to discharge both *C*_c_ and *C*_dl_ completely by introducing an additional switch over *C*_c_. In this case, *C*_c_ and *C*_dl_ are shorted individually and are guaranteed to discharge toward 0 V in pseudosteady state, which would eliminate the offset. However, in this case, it is unclear how the coupling capacitor is contributing to the charge-balancing process: It does not improve *τ*_dis_ and *V*_Cdl_ will have the same response as compared to the circuit without *C*_c_. Furthermore, the additional switch introduces a single-fault device failure risk. Therefore, the advantages of a coupling capacitor are not exploited when *C*_c_ is discharged separately.

This work focused on passive charge-balancing techniques. Active charge-balancing techniques use feedback to bring the electrode voltage back to safe values after a stimulation cycle [15] and can therefore help to overcome the offset problem. However, if these schemes require a coupling capacitor to protect in the event of a device failure, it is important to measure the voltage over the electrodes only and not to include the coupling capacitor. This requires an additional sensing pin if the coupling capacitors are realized using external components. Only then, the feedback mechanism will help to remove the offset.

In this study, only one type of electrode was considered. Smaller electrodes have different impedance levels, and more research is needed to find the pseudosteady-state response in this case. Note that Eq. (4) is only valid under the assumption that *τ*_dis_ ≫ *t*_dis_, which might not be the case for high impedance electrodes. Finally, it would also be interesting to determine the influence of the coupling capacitors in vivo.

## Conclusions

In this work, the influence of coupling capacitors on the charge-balancing properties is studied during neural stimulation. In contrast to what previous work suggests, coupling capacitors were found not to improve the charge-balancing process. Even more so, they introduce an offset voltage in the electrodes, which cannot be removed by conventional means such as passive discharging. The value of the offset voltage depends on the stimulation and electrode parameters. When using coupling capacitors, it is therefore important to ensure that this offset voltage does not exceed any safety boundaries for all possible operating conditions.
